# The Perspectives of Platelet Proteomics in Health and Disease

**DOI:** 10.3390/biomedicines12030585

**Published:** 2024-03-06

**Authors:** Preeti Kumari Chaudhary, Sachin Upadhayaya, Sanggu Kim, Soochong Kim

**Affiliations:** Laboratory of Veterinary Pathology and Platelet Signaling, College of Veterinary Medicine, Chungbuk National University, Cheongju 28644, Republic of Korea; chaudharypreety11@gmail.com (P.K.C.); dr.supadhayaya@gmail.com (S.U.); tkdrnfld@naver.com (S.K.)

**Keywords:** platelet, proteomics, CVDs, cancer, transfusion

## Abstract

Cardiovascular thromboembolic diseases and cancer continue to be a leading cause of death and disability worldwide. Therefore, it is crucial to advance their diagnoses and treatment in the context of individualized medicine. However, the disease specificity of the currently available markers is limited. Based on analyses of a subset of peptides and matching proteins in disease vs. healthy platelets, scientists have recently shown that focused platelet proteomics enables the quantification of disease-specific biomarkers in humans. In this review, we explored the potential of accurate platelet proteomic research, which is required to identify novel diagnostic and pharmaceutical targets by comprehending the proteome variety of healthy individuals and patients for personalized and precision medicine.

## 1. Introduction

Platelets are small anucleated cell fragments that play a central role in regulating thrombosis and hemostasis in the body. They contain more than 1500 proteins, including those involved in platelet activity, and are composed of alpha granules, dense granules, lysosomal granules, and glycogen [1]. Due to platelets’ high granular content of growth factors (GFs), cytokines, and other biological modulators that can respond to a variety of signals and regulate a wide variety of biological processes, including inflammation, angiogenesis, stem cell migration, and cell proliferation, scientific research and technology have recently offered a new perspective on platelets and their functions.

Recently, the objective identification and quantification of the protein profile, the so-called proteome of cells, tissues, or organs, has drawn interest from several sectors as it provides extra useful information for research problems. This tool has been utilized to comprehend disease and to find biomarkers for the prognosis and diagnosis of diseases with various etiologies. Proteomics and platelet biology are sciences that are growing quickly and have great promise. Platelets are thought to act as biosensors for both health and diseases, and their proteome may be used to recognize the telltale signs of both [1]. It is known that platelet production is affected by one’s health status and that they can even take up molecules from nearby cells and release microvesicles into the bloodstream [2,3,4]. Therefore, the clinical management of some pathologies in which platelets play a significant role necessitates the development of alternative therapies, as is the case in patients whose thrombosis–bleeding balance is disturbed, and a proteomics approach may help in the identification of novel targets in such cases. 

It may be possible to find biomarkers that might be employed in early diagnosis, illness prediction, or therapy response by understanding how the platelet protein functions physiologically and how this may be changed in the case of disease. Additionally, because platelets are naturally lacking in a nucleus, proteomics can be one of the most intriguing methods for studying them. Moreover, studying platelet proteomics could make it possible to find new targets for developing individualized treatment plans. On the other hand, platelet proteomics could offer a substantial biomarker-finding tool in other disorders, outside those primarily connected to platelets, given the varied and diversified activities of platelets throughout ontogeny or in inflammation.

## 2. The Principal Role of Platelet: Hemostasis and Thrombosis

Platelets are tiny (2–3 µM in diameter) cell fragments that are the second-most prevalent component of blood circulation, after red blood cells (RBCs). They originate from the cytoplasm of megakaryocytes (MK) present in the lungs and bone marrow. After they become senile, they circulate for 7–10 days in the circulatory system before being eliminated in the spleen or liver [5]. Platelets execute many tasks from primary hemostasis to inflammation depending on their activation. Platelets have various receptors on their surface and biological components kept in various granules that play a role in their activation. Upon platelet activation, the platelet secretes a milieu of physiologically active metabolites and proteins from its granules in a well-regulated manner, and these are effectively transported to their sites of action, which strengthens the coagulative response in a positive-feedback loop [6]. There are three distinct types of granules in platelets: α-granules that consist of a variety of proteins, cytokines, chemokines, and GFs; dense-granules that house small molecules, such as serotonin, adenosine diphosphate (ADP), polyphosphates, and calcium; and lysosomes that house deteriorating enzymes (Table 1) [7]. These contents are secreted through an open canalicular system (OCS), a unique surface-connected network of channels. Toll-like receptor 9 (TLR9), protein disulfide isomerase (PDI), and vesicle-associated membrane protein 8 (VAMP-8) are present in T-granules, and it has been hypothesized that these molecules are attracted to the cell surface and aid in secretion [8]. 

## 3. Activation of Platelets

**Figure 1:**
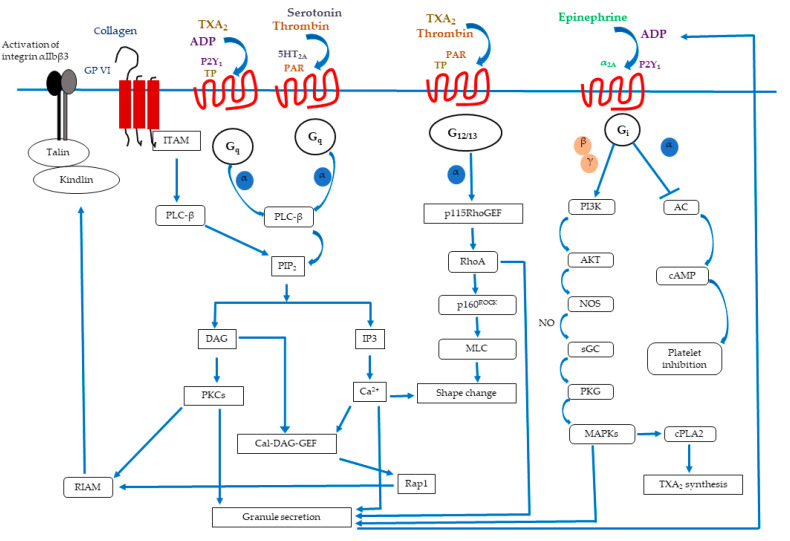
Overall signaling mechanism of platelet activation.

Platelets become activated upon exposure to extracellular matrix proteins including collagen, von Willebrand factor (vWF), or fibronectin when there is any endothelial damage (Figure 1). Normally, endothelial cells help to keep them inactive by secreting prostaglandin I_2_ (prostacyclin, PGI_2_) and nitric oxide (NO), and by expressing CD39 (ectonucleotidase that cleaves ADP/ATP). Platelets react fast to damage to the vessel wall; they adhere to the affected regions and become activated to close the wound [9]. vWF, which is generated from plasma, alters the shape of platelets in high-shear circumstances and enables their binding to the exposed collagen via glycoprotein (GP) VI and αIIbβ3 integrin at the injured region [10]. In addition, the GPIb-V-IX complex is necessary to maintain platelet adherence to the vascular surface under high-shear circumstances [11]. Collagen binding by the integrin α2β1 at low-shear circumstances also plays a significant part in platelet adherence to the damaged endothelium. 

Through GPVI, a member of the immunoglobulin family that is connected to the Fc receptor (FcR), collagen starts the activation of platelets. Immune receptor tyrosine-based activation motif (ITAM), which is present in the cytoplasmic tail of FcR, is phosphorylated by Src kinases [12]. Upon activation, platelets undergo a shapeshift from discoid to a more spherical shape, develop filopodia, and completely expand into lamellipodia. Activated platelets function in a paracrine and autocrine manner to recruit more circulating platelets and further activate after adhesion. Thromboxane A_2_ (TxA_2_) synthesis and the ADP release from dense granules stimulate TxA_2_ and P2Y(_1_ and _12_) receptors, respectively, by coupling to G-protein to facilitate the activation process. 

The second messengers inositol 1,4,5-triphosphate (IP_3_) and diacylglycerol (DAG), which are derived from the membrane phospholipid phosphatidylinositol 4,5-bisphosphate (PIP_2_), are produced when the G-protein and ITAM-coupled receptors are activated. While DAG will activate several protein kinase C (PKC) isoforms, IP_3_ will cause the release of Ca^2+^ from intracellular reserves [13]. Several platelet reactions, including cytoskeletal modifications, integrin activation, and degranulation, are triggered by an increase in Ca^2+^. The guanine nucleotide exchange factor CalDAG-GEFI, in turn, activates the small GTPase Rap1 in response to an increase in Ca^2+^ [14]. Rap1 then attracts talin to the plasma membrane, further activating integrin αIIβ3 as a result. To regulate the additional cytoskeletal remodeling required for complete platelet spreading and clot retraction, active αIIβ3 will convey outside-in signaling upon binding to its ligands (such as fibrinogen, vWF, etc.) [15]. By attaching platelets to one another, this binding also helps to stabilize aggregates. Last but not least, exposed injured tissue releases tissue factor, which encourages the production of thrombin. Thrombin cleaves fibrinogen into fibrin, which will further strengthen aggregation [16]. As a result of secondary hemostasis, actin-myosin platelet retraction mediates the clot’s ultimate stability. However, unbalanced clot formation (thrombosis) could result in the occlusion of the vessels in certain pathological conditions when the balance between the platelet stimulatory and inhibitory pathways is disrupted. This could result in myocardial infarction, stroke, or venous thromboembolism [17]. In conclusion, it is critical to tightly control platelet activation to guarantee a correct platelet functional response and avoid the development of unintended thrombi that might have serious pathological consequences.

## 4. Platelet Proteomics

A proteome is the complete set of proteins expressed within a defined sample under specific conditions that may be highly complex, as the composition depends on the phase, fate, and environment of a cell, as well being modulated by metabolic pathways and post-translational modifications (PTM). In nucleated cells, there are thought to be about 10,000 distinct proteins, among which 2000–6000 proteins have been shown to be analyzed by proteomic assays. Although platelets are anucleated cells, they retain a large portion of their cytoplasmic content from MKs and thus contain RNA, which carries messages for several platelet proteins including chemokines, FcRs, plasminogen activator inhibitor-1 (PAI-1), and PKC (Table 1). Combining proteomics with platelets will help us to analyze the total protein components of platelets under certain conditions.

Theoretically, the total expression of protein-coding genes in platelets is now predicted to be 1400 proteins based on data from the genome-wide platelet transcriptome. As far as we now understand, mitochondrial, metabolic, signaling/adaptor and transcription proteins are notably prevalent in the discovered platelet proteome [18]. A lot of progress has been achieved in the last 10 years in determining the protein makeup of platelets which are newly separated from human blood samples. The number of proteins has grown from 1300 proteins discovered by mass spectrometry and label-free analysis in 2011 to 5400 proteins, of which 3700 have estimated copy numbers [19,20,21]. The 500 proteins with the greatest copy numbers were analyzed, and it was found that proteins involved in signaling, small GTPases, the actin and microtubule cytoskeletons, and α-granules were the most abundant [22]. Numerous virtually intact 20 S and 26 S proteasomes were also found, supporting normal protein degradation in platelets [23]. A study also showed that 80 proteins (9%) associated with plasma proteins and signaling proteins had different abundances in small and big platelets generated from single healthy donors [24].

## 5. Methods of Platelet Proteomic Analysis

Platelet isolation without contamination is a challenging procedure and is performed by the centrifugation of whole blood. Indeed, various factors including the recent administration of a drug (aspirin, prednisolone), quick isolation after blood collection, age, and platelet suspension temperature may modify the protein of platelets [23,25,26,27]. Therefore, various methods are used to prepare the proteomics sample depending upon the purpose of the experiments [28]. The most widely used method is the lysis of platelets to extract the protein in them, and several lysis methods have been used, among which glycerol lysis appeared to be the most reproducible and efficient [29].

Protein separation can be done by two processes: Sodium dodecyl sulfate–polyacrylamide gel electrophoresis (SDS–PAGE) and two-dimensional gel electrophoresis (2-DE). In SDS-PAGE, proteins are separated according to polypeptide size using polyacrylamide gel in which low to medium-size separation occurs [30]. In the high-resolution 2-DE process of protein separation, proteins are separated by their two distinct properties: initially by their isoelectric point and then according to their relative molecular mass [31]. The composition of platelets’ subcellular organelles, such as lipid rafts, membranes, secretory granules, and platelet microparticles, as well as the identification of proteins and the mapping protein phosphorylation of resting and active platelets, have all been studied using this technique. The rough relative measurement and monitoring of platelet differences under various physiological and pathological situations were made possible by further protein comparison staining or pre-labeling of the proteins from various biological samples and mixing them before separation. Mass spectrometry (MS) has increasingly become the method of choice for the analysis of complex protein samples and is currently proteomics’ most important tool, as it measures the femtoliter concentration of protein [32]. For the digestion of protein to generate peptides in MS, enzymatic digestion by trypsin is known to be the best method used [33]. To minimize the complexity of the mixture, high-performance liquid chromatography (HPLC) is used in combination with electrospray ionization coupled to MS. The mass analysis and detection of peptide ions are performed by the MS. MS analysis of the peptides is divided into peptide mass fingerprinting and tandem MS (MS/MS) [34]. MS/MS allows for the sequencing of proteins and peptides, which is why it is an indispensable tool for the recognition of proteins, detection of the site of phosphorylation, structure illustration, and characterization of PTMs [35]. In MS/MS, labeling leucine is performed in protein identification [36]. The advantages and disadvantages of each method of platelet proteomics analysis are listed in Table 2.

To determine the quality of protein, quantitative proteomics can be performed. There are two different approaches for the quantification of protein which are label-free and isobaric labeling (tandem mass taqs or isobaric tags for relative and absolute quantification; iTRAQ). The label-free quantification approach has been the most popular and is the simpler technique, and measures the absolute concentrations of all proteins based on summarized ion counts. After protein biosynthesis, PTM is performed to control multiple biological functions of protein: protein folding, localization, and interaction with other biomolecules [37]. In the case of platelets, PMTs are studied for the phosphorylation sites, ubiquitylation, and proteolysis of proteins, as well as some special interest in platelet activation [34]. Additionally, pathway and network analysis techniques have become increasingly popular, as these aim to identify activated pathways and pathway modules from functional proteomic data [38]. Pathway analysis also helps to organize a long list of proteins in a short list of pathway knowledge maps, which makes the interpretation of molecular mechanisms easier when they are involved in protein alteration and their expression. 

Validation of the proteomics analysis is a crucial step to confirm the data and can be performed by several methods including western blot, ELISA, immunoblotting, and immunoprecipitation [39].

## 6. Platelet Proteome in Health and Diseases

**Figure 2:**
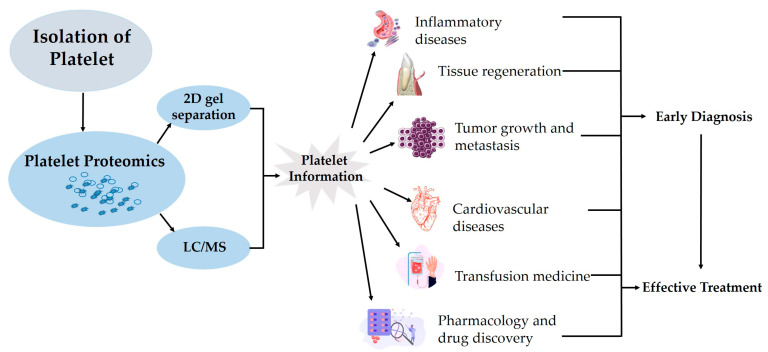
Schematic diagram of platelet proteomics and its application.

When platelets are activated, they release a variety of chemicals that can have an impact on various pathophysiological processes, such as inflammation, tissue regeneration and repair, cancer growth, and cardiovascular diseases (CVDs) (Figure 2). Earlier studies on platelet proteomics, phosphorylation, and other PTMs are done in resting and activated states where the composition and copy numbers of human and mouse platelets are thoroughly described [20,21,40]. Additionally, the “platelet release”, the term for the proteomic composition of the granules released by activated platelets, is described and characterized [6]. A thorough map of human platelets and an examination of inter- and intra-donor variation also revealed that 85% of the platelet proteome is stable [21,41]. Since the fundamentals are already established, this can be used to investigate how various illnesses change platelets and, hopefully, to understand how to target those signaling pathways with drugs. 

CVDs are the primary cause of death in developed countries, and it is well-known that platelets play a significant role in their development. Methods for the detection and prognostication of CVD progression are urgently needed, but the underlying signal transduction is still poorly understood. Acute coronary syndrome and stable coronary artery disease may now be distinguished using comparative proteomics of “platelet releasates” [42]. A study showed that 6 out of 400 proteins that were tested had distinct expression patterns in individuals with acute and chronic coronary syndromes [43]. In a small cohort of 10–30 participants that compared acute vs. chronic coronary syndromes in individuals, it was established that the differently regulated proteins have a role in the cell structure, morphology, and cell assembly processes, all of which are crucial for platelet activation [34]. In particular, signaling, glycolysis, and cytoskeletal-related platelet proteins were shown to be differentially altered in two groups of patients with acute coronary syndrome [44,45]. 

Likewise, in contrast to circulating platelets, gel-based proteomics found a change in 16 platelet proteins including integrin αIIb and thrombospondin-1 collected from the intracoronary culprit site in patients with ST-elevation myocardial infarction (STEMI) [46]. Furthermore, a platelet phosphoproteomic study of STEMI patients showed an elevation in critical tyrosine phosphorylation upon GPVI activation, raising the idea that GPVI might be used as an antithrombotic target in STEMI [46,47]. Platelet releasate from individuals with stable angina pectoris and whole platelets from patients with lupus anticoagulant-related thrombosis showed that only a small number of proteins are changed [48].

According to targeted mass spectrometry, the difference in integrin αIIbβ3 was found to be only 5% for platelets from control participants compared to patients with type I Glanzmann thrombasthenia, a severe bleeding disease [49]. In addition, as compared to control platelets, plasma proteins endocytosed by integrin αIIbβ3 seemed to be downregulated, including fibrinogen, factor XIII, plasminogen, and carboxypeptidase 2B. Quantitative proteomics analysis on platelets from a patient with Scott syndrome, a rare moderate bleeding condition, showed that 134 (6%) proteins were either up- or down-regulated, including the full absence of the phospholipid scramblase anoctamin-6 and low levels of the platelet-morphology-regulating calpain-1 protease [50]. Likewise, in patients with the severe bleeding condition X-linked thrombocytopenia with thalassemia, 83 changed proteins along with cyclooxygenase 1 (COX1) and a number of the cytoskeleton and proteasome proteins were discovered by quantitative proteomics [51]. Additionally, 123 platelet proteins were primarily downregulated in 5 out of 47 gray platelet syndrome patients (a milder bleeding condition) with novel variations in NBEAL2, with the majority being granule-associated and cargo proteins at unchanged mRNA expression levels [52].

Although several studies have looked at the platelets from individuals with cardiovascular conditions, complete platelet proteomic evidence is still lacking. Large-scale validation studies are necessary to determine whether platelet proteomics may be a valuable tool in cardiology treatment and clinical practice, even though prior research has shown that platelet activation varies across certain cardiovascular illnesses. With the most recent technology, it is possible to monitor the protein abundance in “platelet releasates” to assess uneven platelet reactivity and probable future thrombus development.

Some proteomic studies have looked at platelets from individuals with somatic mutations in cancer or genetically less well-defined disorders in addition to uncommon congenital defects. Quantitative proteome analysis research revealed disease regulation by a wide range of platelet proteins from 12 patients with early-stage malignancies, in contrast to healthy participants [53]. The majority of these proteins returned to normal following surgical resection. It has been suggested that the platelet proteome contains variably expressed proteins linked to early-stage cancer, and as a result, platelet proteins are identified as a novel source of potential biomarkers [53]. However, these findings were supported by proof-of-concept research conducted only on a small cohort of patients with lung or pancreatic cancer. Additional focus is required in this area as some of the proteins that are controlled by platelets may serve as biomarkers for certain malignancies.

Platelet function issues also are linked to chronic kidney disease, which can result in bleeding and thrombotic problems, leading to high morbidity and mortality [54,55]. Platelet glycoproteins GPIIb/IIIa, serotonin, and ADP release, as well as problems with the metabolism of arachidonic acid and prostaglandins, are potential contributory factors [54]. Additionally, uremic toxins have been demonstrated to affect endothelial cells, vascular smooth muscle cells, macrophages, and platelets, increasing inflammation and causing platelet activation and aggregation [56]. Due to platelets’ multifactorial nature, disease stage-associated variability, and interpatient variability, unraveling the factors that contribute to platelet dysfunction through proteomics analysis for diagnostic and prognostic interests would substantially aid in the recognition of risk factors and treatment alternatives.

Significantly, the amounts of plasma proteins involved in immunological responses and inflammation were increased, which showed that these patients may also have an immune deficiency. Plasma proteins including fibrinogen and 2-macroglobulin, which are associated with enhanced endocytosis or stickiness of the patient’s platelets, were shown to be raised in the platelet proteome of individuals with progressive multiple sclerosis [57]. Platelet quantitative proteomics found roughly 300 regulated proteins in dengue virus-infected individuals [58]. A total of 360 differently regulated proteins were discovered, among which four of them, PHB, UQCRH, GP1BA, and FINC, were effective in differentiating between patients and healthy controls during the platelet proteomic analysis of patients with mild and severe cognitive impairment in the search for an Alzheimer’s disease biomarker [59]. 

Neutrophil Extracellular Traps (NETs) are formed by neutrophils during the immune response by a controlled cell death process called NETosis and are web-like structures composed of DNA and histones [60]. TLR2 and TLR4 are involved in the activation of neutrophils [61]. Histones within NETs can also activate platelets directly via TLR2 and TLR4, enhancing platelet aggregation and thrombin production [62]. Histones also stimulate the release of vWF from vascular endothelial cells, mediating further platelet adhesion and aggregation. Importantly, platelet activation can cause the dysregulation of NETosis, which can result in immune-mediated scattered microthrombi, hypercoagulability, and tissue damage through the vWF–NETs axis, leading to multiple organ failure and death [63,64]. For example, the vWF–NET axis has been noted to contribute to thrombotic complications in acute ischemic stroke and COVID-19 [65]. It has been observed that NETs cause patients with gastric cancer to have hypercoagulable platelets by upregulating the cell-surface expression of P-selectin and phosphatidylserine [66,67]. Malignant tumors may also trigger immunothrombosis by stimulating neutrophils and/or platelets, which is followed by the formation of a NET [68]. Furthermore, Guglietta et al. provided a link between NETs and platelets in an animal model of small intestinal tumors [69]. There is evidence that the interaction between platelets and NET causes autoimmune diseases like systemic lupus erythematosus by affecting coagulation [70]. It has now been demonstrated that type 1 diabetes and the platelet–neutrophil interaction are related [71]. The pancreas of non-obese diabetes mice showed a correlation between an increase in platelet–neutrophil aggregates in the circulatory system and NET markers, indicating that platelets may stimulate neutrophils for transmigration into islets, which is followed by NETosis and islet destruction. The TLR4–ERK5 platelet axis has been shown to facilitate NET formation in the lung and further promote metastasis [72]. Taken together, the TLR-mediated vWF–NET signaling pathway plays an important role in immune response and is linked to the pro-thrombotic state in various diseases. The detailed analysis of proteins involved in vWF–NET axis-mediated thrombosis and inflammation may be a valuable tool in identifying novel biomarkers and therapeutic targets in CVDs and other diseases associated with thrombosis.

## 7. Platelet Proteomics in Transfusion Medicine

A long-standing problem is how to optimally store platelets to preserve their functions after transfusion [73]. Proteomics is being actively used to describe temperature-induced platelet changes. According to transcriptome-based research, freshly separated platelets have a limited potential for protein synthesis and are continuously degrading RNA species [74]. Frequently, platelet concentrates used in transfusions may be kept in storage for a few days before the platelets begin to lose their functional characteristics, a condition known as platelet storage lesion [75]. Numerous investigations on the protein alterations of aging platelets have been conducted to determine the source of this lesion. In an early study, the majority of the 2900 identified proteins were found to have new N-termini, which showed that platelet storage included significant proteolytic processing [76]. Endocytosis- and cytoskeleton-related proteins were shown to alter with platelet age to enrich younger circulating platelets in a platelet apheresis intervention program [77]. According to two quantitative proteomic investigations, changed proteins in particular had a role in degranulation as the storage duration increased [78,79]. Due to the stimulation of glycoprotein shedding, platelets kept at 2–6 °C were shown to exhibit lower levels of glycoproteins and higher amounts of surface activation indicators, although their viability was unaffected [80]. In the wake of the transfusion of aging platelets, several organizations are looking for proteins present in the platelet that might justify harmful transfusion responses that harm the health of patients. In terms of pathogen inactivation, the exposure of concentrated platelets to riboflavin and ultraviolet light for two days led to the production of reactive oxygen species, which led to a slight increase in the number of oxidized peptides when compared to the 18% of the 9400 identified platelet peptides that were already oxidized [81,82]. Age-related upregulation of proinflammatory cytokines (CCL5, PF4) and metabolic proteins (such as glycolysis and lactate synthesis) was seen in proteomic investigations on extracellular vesicles produced by aging platelets [83].

As technology advanced, proteomic research using label-free quantification showed that prolonged storage for 13–16 days decreased the levels of proteins involved in platelet degranulation, secretion, and exocytosis while increasing the levels of 2-macroglobulin, glycogenin, and Ig chain C region [78]. Wang et al. discovered that varied storage temperatures resulted in various PSLs after comparing the proteomic signatures of platelets kept at 22 °C, 10 °C, and 80 °C. While cold storage affects SNARE interactions in vesicular transport and vasopressin-regulated water reabsorption, the storage duration mostly affects endocytosis, Fc gamma R-mediated phagocytosis, and actin rearrangement [79]. Concentrated platelets for customized transfusions may become available in the future of precision medicine.

## 8. Hurdles in Accurate Platelet Proteome Research

While studying platelets by proteomics may appear simple and straightforward, there are several issues, mostly with the quality control of samples used in this proteomics research. The use of high-throughput proteomics to study platelet biology raises several issues, as accepted by all industry professionals. These range from the collection of blood samples, such as the isolation method, use of anticoagulant, and sample processing, to components of mass spectrometry technology and data analysis, such as protein detection low-abundance, modifiable protein abundance, etc.

Washed platelets are often employed in platelet proteome studies today. Reliable proteome analysis depends on the platelet purity, quantity, and activation state. Although washed platelets have been isolated in most studies with a high purity (99–99.99%), the presence of proteins from plasma, RBCs, and white blood cells (WBCs) cannot be totally ruled out [18]. The platelet separation process is often built upon a series of low-speed centrifugation stages that separate whole blood into distinct blood components depending on their densities while leaving the tiny, light platelets floating in the liquid plasma [84,85]. However, because platelets in direct contact with blood plasma have a prolonged open canicular system, leftover plasma proteins are always present in the majority of listed platelet proteomes [18]. Although the ability to separate platelets from whole blood has increased throughout the years, earlier studies with platelet proteomics demonstrated a broad range of platelet purities, platelet concentrations, and protein use quantities per sample under analysis. In the first proteomics investigation on mice, protein abundance patterns were examined along several purification processes to separate real platelet proteins from impurities in plasma, WBCs, and RBCs [40]. The study showed that there can be the presence of more than 200 impurities (mostly RBC components or extremely abundant plasma proteins such as apolipoproteins, and complement factors due to the clustering of proteins at various purification stages) [40]. 

It was claimed that the OptiPrepTM density gradient centrifugation method could recover more than half the population of platelets with a 99.99% platelet purity and little WBC contamination, but it has not been used for proteomic investigations. Microfluidic platelet preparation or a completely automated method that offers a high yield and purity (>99%) with the reduced activation of platelets are suggested to be used in place of centrifugation methods for analyzing the platelet transcriptome [86]. 

For high-throughput research, additional controls should be created to guarantee full platelet lysis and digestion, low peptide loss during the preparation of samples, and automation for the analysis of a large number of samples. In the laboratories working with platelets’ biological and translational applications, quality control methods of mass spectrometry-based proteomics data acquisition must be established. For example, a cloud-based quality control system or web-based apps can be applied [86,87,88]. Label-free analysis, which allows for infinite numbers of samples to be compared at the level of the proteome, can be done for greater throughput applications [78]. 

Searching known databases of fragmentation spectra is the most often utilized method for proteomic data analysis. It provides a useful summary of current bioinformatics approaches for the identification and quantification of proteins [89]. Data normalization, which involves applying adjustments in accordance with predetermined standards to eliminate inconsistent data points followed by statistical testing/screening for false discovery rates, is a crucial stage in the analysis. A changing protein abundance should not be the reason for the apparent regulation of a phosphorylation site in phosphoproteomics [90]. Special methods must be used to determine a phosphorylated peptide’s site of phosphorylation and the kinase that is involved with platelet function [91].

One of the major drawbacks that has been observed in several studies is the inclusion of just a few platelet samples for comparison. Because of this, even when comparing healthy individuals to those who are sick, it has become challenging to make conclusions about inter-subject variances. Other technical drawbacks include inadequate protein abundance that is associated with inadequate peptide coverage, difficult spectral data processing, missing hydrophobic peptide sequences, and uncertain functions for several newly found proteins [92]. Therefore, the quantity of samples is another aspect that cannot be overlooked in platelet proteomic research. Strategies must be established in the future that allow for the use of only a few microliters of blood for platelets-based proteome study. 

Since many studies only use a small number of samples and mass spectrometry proteome analyses are still expensive, it is difficult for institutions to conduct research with the ideal number of samples needed for careful data interpretation. Proteomics analysis is complicated and needs educated employees, which prevents the widespread use of the technology at diagnostic clinical laboratories. The equipment may not be inexpensive for clinical institutions. This raises questions about how well platelet proteomics may be applied in the therapeutic setting. It is recommended that standard protocols and areas of concern need to be created for platelet preparation, sample processing, and data analysis in order to increase the reproducibility of platelet research.

Most importantly, PTMs are another big hurdle in studying platelet biology. PTM is defined as the addition, subtraction, exchange, or rearrangement of functional groups to the side chains of amino acids and the N- and C-termini of proteins. After biosynthesis, proteins are subjected to PTMs, which are known to regulate a variety of biological processes, including protein activity, folding, localization, and interactions with other biomolecules [93]. Although more than 400 PTMs have been characterized, the full extent of their physiological functions is yet unknown. Phosphorylation, ubiquitylation, and proteolysis are the PTMs for platelets that have been extensively researched, while glycosylation, acetylation, and palmitoylation are given little consideration. The interaction of platelet PTMs during activation is also particularly intriguing.

Although there will be a lot of difficulties in making this shift, the development of proteomics as a basic tool for platelet research and moving this field from the discovery stage into the biology and preclinical application stage is important. In summary, for the field to advance, standardized criteria that enhance the repeatability of platelet preparation, proteomic sample processing, and complicated data analysis across laboratories should be implemented. 

## 9. Is Clinical Translation between Mouse Platelet Proteome to Human Clinical Studies Possible?

Preclinical models have enabled a wide range of clinical uses, including surgery, vaccine development, illness detection, and therapy, among others. According to the thorough understanding thus far drawn from interspecies investigations, conclusions gained from animal research cannot be casually extrapolated to humans. Nevertheless, some research topics need the use of animal preclinical models, where researchers may phenocopy human illnesses, pathologies, or diseases in order to better understand the process underlying these qualities and test possible innovative therapies. 

Human and mouse platelets share a substantially conserved proteome, thus providing evidence for the application of the proteomics technique in both intra- and inter-species fields [94]. This is also supported by an increasing number of preclinical or clinically applicable investigations in humans. Additionally, the similarity of the physiological, anatomical, and genetic characteristics between mice and humans justifies the use of mice as preclinical models, despite the evident differences between the two species [95]. Moreover, we have the ability to modify them genetically and physiologically. Therefore, although there are differences between the platelet formation processes in humans and mice, it is possible to study the megakaryopoiesis, thrombopoiesis, and platelet function of humans using mice as a model [95,96]. Murine preclinical models can enable us to illustrate how different proteins, including transcription factors, receptors, signaling molecules, hormones, cytokines, etc., function pathophysiologically in humans [97,98].

## 10. Conclusions and Future Perspectives

Myocardial infarction, ischemic stroke, and pulmonary embolism are just a few examples of cardiovascular thromboembolic diseases that continue to be the leading causes of death and disability in the world. Therefore, it is crucial to advance diagnoses and treatment in the context of individualized medicine. The disease specificity of currently available indicators is restricted, despite the fact that traditional platelet-activation markers are sensitive in detecting excessive or faulty activation states. Recent studies of platelet proteomics signatures, in contrast, demonstrate an enhanced disease specificity. Based on analyses of peptides and matching proteins in disease vs. healthy individuals, it has recently been shown that focused human platelet proteomics enables the quantification of CVD and other thrombotic disease biomarkers. In a customized and precision medicine scenario, it will become more critical to analyze the individual diversities in the proteomes of healthy people and patients. 

Although the studies on the subject must be further validated by various independent investigations, the recent advances in mass spectrometer technology have made it possible to analyze numerous previously unidentified and newly discovered proteins quantitatively and to reveal the intricate phosphorylation patterns of proteins, many of whose functions are still unknown. The discovery of novel platelet proteins as possible biomarkers for diseases would be another breakthrough in the proteomic discipline. Some of the altered proteins that are indicative of an increased risk for CVDs and other platelet-associated disease states, reflecting alternations in platelet function and signaling pathways, are listed in Table 3. 

Despite wide variations in mass spectrometry and spectrum analysis techniques, the exact composition of the ‘typical’ human platelet proteome is still unknown. The attainable human platelet proteome will need to be defined through a coordinated multi-laboratory effort. The manner and purity of sample preparation, including the platelet concentration, activation state, and all sample processing, differ significantly between the studies done to date. In order to compare new research results more effectively, inter-laboratory standardization is expected to be required. Independent methods to confirm conclusions about protein up- or down-regulation have led to the inconsistency and non-repeatability of the research results. 

The physiological roles in platelets of many proteins of putative biomarker importance remain unknown, and more complex protein function studies than general pathway studies are needed (e.g., using Gene Ontology). This objective may be accomplished with the use of a recent categorization scheme of all proteins predicted to be or present in platelets. Mass spectrometry can be substituted by less expensive, immune-based, or flow-cytometry techniques in bigger/clinical research if a biomarker is confirmed.

Low sample sizes have prevented published studies of patients from examining common intersubject factors, including blood cell characteristics, gender, age, and health history. The simultaneous comparison of several platelet samples is possible thanks to new high-throughput analytic techniques that combine label-free quantification approaches with data-independent acquisition, which is necessary for these clinically pertinent concerns.

Detailed information regarding protein distributions in healthy volunteers and patients will be provided by quantitative proteomics research. The phosphorylation patterns of platelets will also be helpful in comprehending platelet activation and finding potential new treatment approaches. Beyond the conventional depiction of linear pathways, a greater knowledge of platelet signaling will be possible thanks, in particular, to quantitative phosphoproteomic research. Over the past decade, although these unique technical approaches have and will continue to produce important discoveries, signaling has proven to be much more dynamic than expected in comparison to existing techniques. To identify previously unidentified post-translational changes, the unsupervised elucidation of platelet signaling using artificial intelligence may become increasingly significant in the future. In order to merge traditional biochemical knowledge with unexpected discoveries from big data methods, fresh data analysis strategies are needed to handle the enormous quantity of data generated by quantitative proteomics investigations. 

## Figures and Tables

**Table 1:** Various proteins present in platelet granules.

Granule	Type	Contents	Role
**α-granules**	**Adhesive proteins**	P-selectin, Fibrinogen, von Willebrand factor, Fibronectin, Thrombospondin-1/2, Laminin-8, Vitronectin	Promoting adherence of WBCs to activated platelets and endothelium. Promotion of leukocyte adherence to activated platelets and endothelium Binding to GpIIb/IIIa receptors, factor VIII, integrin α5β1, αvβ3, β1, αIIβ3, αvβ3, α3β1, and α6β1, and uPAR
	**Growth factors**	Epidermal-growth factor, Insulin-like-growth factor, Hepatocyte-growth factor, Platelet-derived-growth factor	Stimulating/inhibiting the proliferation of fibroblasts, epithelial cells, and smooth muscle cells. The major mediator of growth hormone-stimulated somatic growth and growth hormone-independent anabolic responses. The primary mediator of growth hormone (GH)-stimulated somatic growth and GH-independent anabolic responses Metabolic flux of glucose in different insulin-sensitive cell types; plays a key role in β-cell homeostasis. Metabolic flux of glucose in various types of insulin-sensitive cells; plays a key role in the homeostasis of beta cells
	**Angiogenic factors**	Growth factor from vascular endothelium, Platelet-derived growth factor, Fibroblast	Enhance the proliferation, migration, survival, and invasion of endothelial cells Enhance permeability of existing vessels, forming a lattice network for endothelial cell migration, chemotaxis, and homing of bone marrow-derived vascular precursor cells. Improvement of the permeability of existing vessels, formation of a lattice network for the migration of endothelial cells, chemotaxis, and homing of vascular progenitor cells derived from the bone marrow Modulating the proliferation and recruitment of perivascular cells Activation of a serine-rich protein or serine-rich phosphorylating kinase network regulating alternative splicing of vascular endothelial growth factor receptor 1 (VEGFR1) in endothelial cells.
	**Chemokines**	CXCL8/7/1/5/2/6/12	Activating and recruiting neutrophils
		CCL5/3/2/7	Helps in the recruitment of basophils, macrophages, monocytes, eosinophils, polymorph nuclear WBCs, and neutrophils
		IL1β	Recruitment and activation of WBCs
	**Clotting factors**	Factor V	Cleavage of prothrombin to thrombin
		Protein S	Anticoagulant by inhibition of factor IXa
		Factor XI	Hemostasis by activating factor IX
		Factor XIII	Fibrin network stabilization
		Kininogens	Activating factor XI
		Plasminogen	Fibrinolysis by means of binding to the fibrin clot
	**Integral membrane proteins**	Integrin αIIbβ3, GPIba-IX-V, GPVI, TLT-1, P-selectin	Activation of platelets, adjacent platelets aggregation, formation of thrombus. Plays a role in inflammatory insult-induced bleeding. Promotes the adhesion of WBCs to activated platelets and endothelium
	**Immune mediators**	Complement C3/C4 precursor Factor D/H, C1 inhibitor, Immunoglobulins	Triggers inflammation, phagocytosis, cell lysis, and cell activation by cleaving to C3a and C3b by intruders Inhibition of activation of early clotting proteins and the classical complement pathway Antigenic binding and neutralization
	**Protease inhibitors**	α2-antiplasmin, PAI-1, α2-antitrypsin, α2-macroglobulin, TFPI, C1-inhibitor	Inhibition of plasminogen binding to fibrin and fibrin cross-linking Binding to and inhibition of tissue-type plasminogen activator and urokinase-type plasminogen activator Anti-inflammatory properties through the destruction of major proteases Binding of foreign peptides, acting as a humoral barrier against pathogens Blocks the initial steps of the extrinsic coagulation pathway by inhibiting Factor Xa and Factor VIIa Inhibits activation of early coagulation proteins and the classical complement pathway
	**Proteoglycans**	MMP2/9	Degradation of collagen, elastin, fibronectin, gelatin, and laminin and remodeling of the extracellular matrix
**Dense granules**	**Amines**	Serotonin, Histamine	Minimize blood loss by inducing constriction of injured blood vessels and enhancing platelet aggregation Provides aggregation and immunological stimuli
	**Bivalent cations**	Ca^2+^, Mg^2+^	
	**Nucleotides**	ATP, ADP, GTP, GDP	
**Lysosome granules**	**Acid proteases**	Cathepsin D and E, Carboxypeptidases (A, B), Prolinecarboxypeptidase, Collagenase, Acid phosphatase, Arylsulphatase	
	**Glycohydrolases**	Heparinase, β-*N*-acetyl-glucosaminidase	

**Table 2:** Advantages and disadvantages of gel-based and gel-free platelet proteomics analysis.

	Gel-Based (2D)	Gel-Free (LCMS/MS)
**Advantages**	Detection of isoforms on protein level Relative quantification	High sensitivity High throughput High dynamic range PTMs site location Precise quantification Application in clinics
**Disadvantages**	Low throughput Low sensitivity Low protein coverage Time-consuming sample processing Limited detection of hydrophobic proteins	Expensive analytical equipment Sophisticated data analysis

**Table 3:** Various key platelet protein biomarkers involved in CVDs and other platelet-associated disease conditions.

Biomarker	Disease Conditions	References
**Podoplanin**	Tumor-induced platelet activation and tumor metastasis and invasion.	[99,100,101,102,103,104]
**CD40 ligand**	Acute coronary syndromes, coronary revascularization procedures, atherosclerosis, and inflammatory processes.	[105,106,107]
**Platelet-derived growth factors** (**PDGFs**)	Gliomas, sarcomas, leukemias, and epithelial cancers.	[108,109]
**P-selectin**	Coronary heart disease, hypertension, arterial fibrillation, congestive heart failure, stroke, atherosclerosis.	[110,111,112,113,114]
**Glycoprotein IIb/IIIa**	Platelet aggregation, thrombosis, hemostasis, carotid atherosclerosis, and diabetes.	[115,116,117,118]
**Thrombospondin**	Myocardial infarction, heart failure, coronary artery disease, coronary heart disease, abdominal aortic aneurysms.	[119,120,121,122]
**Advanced glycation end products**	Peripheral artery disease increases thrombotic effect in diabetes and coronary heart diseases.	[123,124,125]
**Troponin**	Myocardial infarction, heart failure, arterial fibrillation, Takotsubo cardiomyopathy, stroke, atherosclerosis.	[126,127,128,129,130],
**Signal transducer and activator of transcription**	Chronic inflammation, osteosarcoma, and prostate cancer.	[131,132]
**Vascular endothelial growth factor**	Breast cancer progression, invasion, and migration, angiogenesis.	[133,134,135]
**β2-Glycoprotein I**	Autoimmune condition antiphospholipid syndrome, thrombosis.	[136]
**Oxidized LDL receptors**	Atherosclerosis.	[137]
**Vasodilator stimulated phosphoprotein**	Metastasis in colorectal cancer.	[138]
**Myeloperoxidase**	Atherosclerosis, coronary artery disease, myocardial infarction, heart failure, inflammation, colon cancer, breast cancer.	[139,140,141]
**RANTES**	Acute coronary syndrome, atherosclerosis, inflammation. Development and progression of atherosclerosis, inflammation, thrombosis, diabetes, myocardial infarction, and atherothrombosis.	[142,143,144,145,146,147,148,149,150,151,152]
**Platelet factor 4**	Liposarcoma, mammary adenocarcinoma, and osteosarcoma, inflammation, atherosclerosis, myocardial infraction.	[153,154,155,156]
**VWF**	Atherosclerosis, hepatic carcinoma, hemostasis and thrombus formation.	[157,158,159,160]
**Beta amyloid precursor protein II**	Alzheimer’s disease.	[161,162,163]
**IL-1B**	Atherosclerosis, inflammation, diabetes, coronary heart disease, stroke, peripheral vascular disease.	[164,165,166,167]
**Autoantibody against platelet protein**	Immune thrombocytopenia.	[168]
**Facotor XII**	Coronary heart disease, atherosclerosis, ischemic and hemorrhagic stroke, myocardial infraction.	[169,170,171,172]
**ADAMTS13**	Thrombotic microangiopathies, Thrombotic thrombocytopenic purpura, hepatocellular carcinoma, peripheral arterial disease, coronary heart disease, stroke, heart failure, myocardial infarction, liver cirrhosis.	[158,173]
**Neutrophil extracellular traps** (**NETs**) **interacting protein**	Autoimmune and inflammatory disorders, atherosclerosis, thrombosis.	[174,175,176,177]
**Neutrophil elastase**	Colorectal cancer, gastric cancer, pulmonary arterial hypertension.	[178,179,180]
**Citrullinated histones**	Thrombosis, inflammation, thromboembolism.	[181,182]
**ERK5**	Inflammation, atherosclerosis, hypertension.	[183,184,185]
**Autotaxin**	Alzheimer’s disease, ischemic dilated cardiomyopathy, calcified aortic valve stenosis.	[186,187]
**Cyclooxygenase**	Atherosclerosis, aneurysm	[188,189]
**Platelet-derived microvesicle**	Development and progression of atherosclerosis, inflammation, thrombosis, diabetes, myocardial infarction, and atherothrombosis.	[145,146,147,148,149,150,151,152]
